# A dataset of direct observations of sea ice drift and waves in ice

**DOI:** 10.1038/s41597-023-02160-9

**Published:** 2023-05-03

**Authors:** Jean Rabault, Malte Müller, Joey Voermans, Dmitry Brazhnikov, Ian Turnbull, Aleksey Marchenko, Martin Biuw, Takehiko Nose, Takuji Waseda, Malin Johansson, Øyvind Breivik, Graig Sutherland, Lars Robert Hole, Mark Johnson, Atle Jensen, Olav Gundersen, Yngve Kristoffersen, Alexander Babanin, Paulina Tedesco, Kai Haakon Christensen, Martin Kristiansen, Gaute Hope, Tsubasa Kodaira, Victor de Aguiar, Catherine Taelman, Cornelius P. Quigley, Kirill Filchuk, Andrew R Mahoney

**Affiliations:** 1 Norwegian Meteorological Institute, IT Department, Oslo, 0313 Norway; 2 Norwegian Meteorological Institute, R&D Deparment, Oslo, 0313 Norway; 3 University of Oslo, Deparment of Geosciences, Oslo, 0313 Norway; 4 University of Melbourne, Department of Infrastructure Engineering, Melbourne, 3010 Australia; 5 University of Alaska Fairbanks, College of Fisheries and Ocean Sciences, Fairbanks, 99775 USA; 6 C-CORE, Captain Robert A. Bartlett Building, Morrissey Road, St. John’s, Newfoundland and Labrador, A1B 3X5 St. John’s, Canada; 7 The University Centre in Svalbard, Arctic Technology Deparment, Longyearbyen, 156 N-9171 Norway; 8 Institute of Marine Research, Fram Centre, P.O. Box 6606 Langnes, 9296 Tromsø, Norway; 9 The University of Tokyo, Graduate School of Frontier Sciences, Kashiwa, 277-8561 Japan; 10 Japan Agency for Marine-Earth Science and Technology, Yokosuka, 237-0061 Japan; 11 UiT The Arctic University of Norway, Department of Physics and Technology, 9037 Tromsø, Norway; 12 Norwegian Meteorological Institute, R&D Department, Bergen, 5007 Norway; 13 University of Bergen, Geophysical Institute, Bergen, 5007 Norway; 14 Environment and Climate Change Canada, Environmental Numerical Prediction Research Department, Dorval, QC K1A 0H3 Canada; 15 University of Oslo, Department of Mathematics, Oslo, 0313 Norway; 16 University of Bergen, Department of Earth Science, Bergen, 5007 Norway; 17 University of Oslo, Department of Physics, Oslo, 0313 Norway; 18 Arctic and Antarctic Research Institute (AARI), St, Petersburg, Russian Federation; 19 University of Alaska Fairbanks, Geophysical Institute, Fairbanks, 99775 USA

**Keywords:** Physical oceanography, Cryospheric science

## Abstract

Variability in sea ice conditions, combined with strong couplings to the atmosphere and the ocean, lead to a broad range of complex sea ice dynamics. More *in-situ* measurements are needed to better identify the phenomena and mechanisms that govern sea ice growth, drift, and breakup. To this end, we have gathered a dataset of *in-situ* observations of sea ice drift and waves in ice. A total of 15 deployments were performed over a period of 5 years in both the Arctic and Antarctic, involving 72 instruments. These provide both GPS drift tracks, and measurements of waves in ice. The data can, in turn, be used for tuning sea ice drift models, investigating waves damping by sea ice, and helping calibrate other sea ice measurement techniques, such as satellite based observations.

## Background & Summary

Sea ice is a major component of the global Earth ecosystem: it covers around 7% of the global oceans, averaged over a year^1^, and strongly modulates the coupling between the ocean and the atmosphere, as well as the global energy balance of the polar regions^2,3^. For example, a strong nonlinear coupling exists between ocean conditions and sea ice extent in the Arctic, due to the effect of ocean waves^4^. Indeed, as sea ice extent decreases in the Arctic basin, a new area of open ocean emerges between the polar ice cap and the surrounding continental landmasses. This, in turn, creates a new region of fetch where waves can grow larger before interacting with the sea ice. As a consequence of these larger waves, more sea ice breaks which also accelerates melting^5,6^, leading to a positive feedback loop. Similar couplings exist due to, for example, the albedo differences between open ocean and sea ice^7^. These changes are exacerbated by the polar amplification of climate change^8,9^.

Unfortunately, accurately predicting sea ice dynamics under the influence of waves, winds, and currents, remains a challenging task^4,10–18^. This is due to the diversity of both sea ice and weather conditions that can be found in the polar regions, as well as the wide range of physical mechanisms at stake, including sea ice breaking, melting, freezing, collisions between ice floes, drifting, wave diffraction, reflection, viscoelastic effects in the ice, and turbulence in the water under the ice^12,19–30^. This makes the development of fully coupled ocean-atmosphere-sea ice-waves models a challenging task^31–34^.

As a consequence, careful validation and calibration of sea ice models over a wide range of sea ice conditions is critical. Unfortunately, calibration data about sea ice drift and waves in ice are scarce, especially in the Marginal Ice Zone (MIZ), i.e. the sea ice area closest to the open water, which is most influenced by waves from the open ocean^35^. Gathering *in-situ* data in this region is made challenging due to the statistically short lifetime of instrumentation under dynamic sea ice conditions, while remote sensing is, at present, challenging to use in a systematic way, and not yet able to provide all the information needed to calibrate models, especially regarding wave properties^36^.

**Fig. 1:**
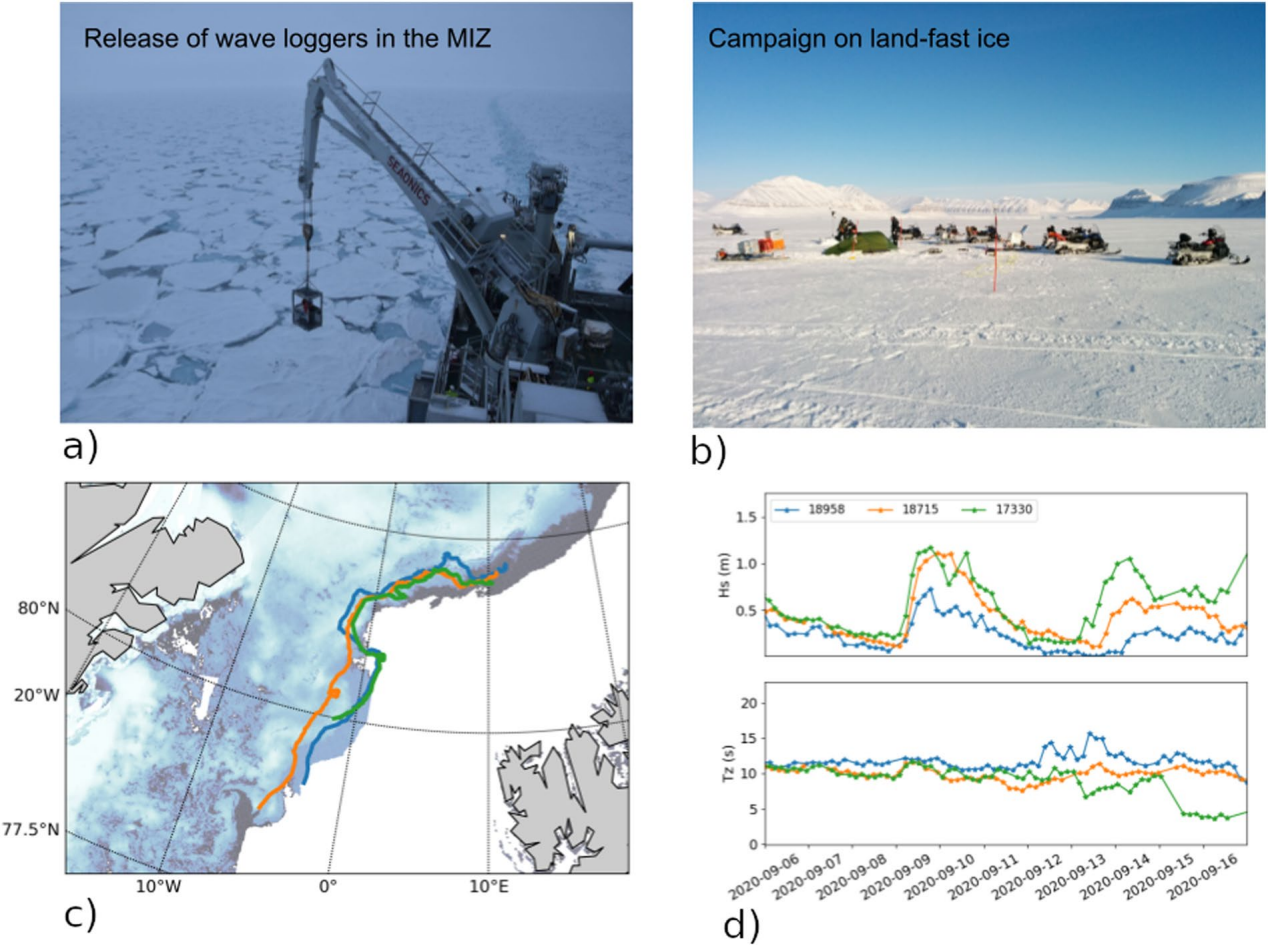
Sea ice can come in many forms, from fields of small broken floes (**a**), to continuous pack ice, to landfast ice (**b**). This has a strong, complex influence on wave damping and sea ice drift. To illustrate the effect of sea ice on wave damping, we show the map presenting Sea Ice Concentration (SIC, **c**) around 3 drifters corresponding to the 2020-07 deployment on 2020-09-16, and the corresponding drifter trajectories. The drifters are at different depth into the Marginal Ice Zone (MIZ), which implies different degrees of frequency-dependent significant wave heigth damping and associated peak frequency shift as higher frequencies in the wave spectrum are more heavily damped than low frequency components and the dominant wave period *T*_*z*_ therefore increases deeper into the sea ice (**d**). The field data released in this manuscript features many similar events, that can be used to tune numerical models.

To help close the gap in sea ice data in the MIZ and other regions affected by waves propagating from the open ocean, we have focused on deploying instruments in both the Arctic and Antarctic. These instruments collect both drift information and wave energy spectrum. Two situations are of particular interest. The first corresponds to sea ice in the MIZ, where broken ice floes drift and break up under the influence of currents, winds, and waves. In these conditions, we usually deploy several instruments perpendicular to the MIZ edge, going successively deeper into the ice, so that we can quantify the effect of sea ice concentration on drift patterns, as well as wave damping into the MIZ, as illustrated in Fig. 1. The second canonical situation of interest is continuous pack ice or landfast ice just outside of the MIZ. Remotely forced waves that are only partially damped in the MIZ can reach this region. This, in turn, can lead to breakup of the continuous ice, which can drift away towards the open ocean where it breaks down further and melts^5^. In these conditions, we deploy instruments close to the limit between the MIZ and the continuous ice, in order to study the relationship between incoming wave energy and continuous ice breakup.

In the present dataset, we release data collected over the last 5 years by 13 groups, which corresponds to 15 deployments, involving a total of 72 instruments, covering both the Arctic, and several regions in the Antarctic. These data can, in turn, be used for investigating the effect of winds, currents, and waves on sea ice, to both identify physical processes, and help further develop and calibrate fully coupled models, in particular, regarding sea ice drift, waves in ice, and sea ice breakup. Moreover, these *in-situ* data are ground observations necessary to help calibrate satellite derived algorithms used in remote sensing of the sea ice.

## Methods

In the course of the data collection, several instrument models have been used. These are, the instrument “v2018”^37^, the instrument “v2021”^38,39^, the Sofar Spotter buoy^40^, commercial Global Positioning System (GPS) drifters with Iridium communication ability, and the Ice Wave Rider (IWR)^41^. All instruments use GPS to measure geographical location. The instrument v2018, v2021, and IWR, use acceleration measurements from inertial measurement units to measure wave motion. By contrast, the Sofar Spotter uses GPS to measure the wave motion.

In the following, we describe how measurements of drift and waves are performed, and we pinpoint differences between the instruments when relevant. The methodology used by the Sofar Spotter buoy is only partially known, as this is a close source, black box instrument. By contrast, the firmware and post processing code for the v2018, v2021 are fully open source, while the IWR is simply performing logging of an inertial measurement unit, with known configuration^41^. Therefore, and in addition to the detailed self-contained methodology description we provide here, the open source codes (available Github at https://github.com/jerabaul29/LoggerWavesInIce_InSituWithIridium and https://github.com/jerabaul29/OpenMetBuoy-v2021a, respectively) can also be used as a source for technical details around the methodology used by the instruments v2018 and v2021. These data cover locations and times when no other direct, *in-situ* observations that we know of are available, and are, therefore, potentially useful for a wide range of sea ice and marginal ice zone studies.

### Drift measurements using the global positioning system

Drift tracks are measured using a GPS receiver. No processing of any form is applied on the output produced by the GPS module. The GPS module has built-in hardware and software that ensure that only valid GPS positions are produced.

- In the case of the v2018, the full GPS National Marine Electronics Association (NMEA) output string is transmitted through Iridium. In addition, all the data collected by the instrument v2018 are stored on an internal SD card, so that, if the instrument v2018 is collected at the end of the deployment, the same GPS data are available there. The GPS measurements are performed with a period of approximately 3 hours.
- In the case of the v2021, only the UTC date and time as well as the latitude and longitude are transmitted. Other information is not transmitted, to save memory and cut satellite transmission costs. However, a higher GPS position sampling rate is applied, typically every 30 minutes.
- Similarly, the Sofar Spotter transmits GPS UTC time, latitude and longitude as a part of its iridium communications.
- The IWRs log GPS UTC time and geographic coordinates hourly to the internal SD card, without performing satellite transmission. Such routine is possible since IWRs deployments are either in ice camps or on shore-fast ice, and the data are retrieved together with the instruments at the end of the deployments.
- Commercial drifters also transmit GPS UTC time, latitude, and longitude.

### Wave measurements using GPS

The Sofar Spotter uses GPS data in order to directly measure 2D wave surface displacement at 2.5 Hz in the North-South and East-West directions. These data are used to compute the wave properties assuming that the underlying signal corresponds to the circular wave orbital velocity^40^. According to the datasheet delivered by Sofar, the typical resolution for significant wave height measurements is +/−2cm, depending on the conditions and sky view. The details of the implementation regarding how the raw data are filtered and how the wave spectrum is computed are, as far as the authors know, not available for this commercial close source instrument. The produced outputs include both wave statistics (significant wave height, and various wave period estimates), full wave spectra, and directional information (some of these outputs can be switched on and off individually of each other). The Sofar Spotter is considered a well-tested instrument, with several thousands units deployed according to the manufacturer, and it has been used in a number of peer reviewed measurement campaigns in the sea ice^42–45^.

### Wave measurements using inertial measurement unit data

The instruments v2018, v2021, and IWR, use Inertial Measurement Units (IMUs) to measure the wave motion. In the following paragraphs, we outline the main lines of the data acquisition and the wave processing algorithms, though the reader curious of the exact, in-depth technical details regarding the open source instruments, is referred to the technical papers^37,38,41^ that go deeper into the exact implementation, or to the code implementing the data processing (see the code released at https://github.com/jerabaul29/LoggerWavesInIce_InSituWithIridium, https://github.com/jerabaul29/OpenMetBuoy-v2021a).

### Measurement of the wave vertical acceleration

The first step in measuring waves is to record the wave signal, i.e acceleration due to the wave motion. A duration of 20 minutes is used as the default time segment used to produce one wave spectrum and its associated statistics, and we used a wave acceleration sample frequency of 10 Hz unless specified otherwise. In order to gather this wave signal:

- In the case of the v2018^37^, all the data collection and Kalman filtering process is implemented by the thermally calibrated Vectornav VN100 Inertial Measurement Unit (IMU) that is used in the instrument^46–48^. The VN100 is an industry-grade IMU, and the details of the Kalman filter and signal processing algorithms running on it are proprietary and close source. The VN100 IMU outputs a number of variables, including accelerations measured in the Earth frame of reference (North, East, Down directions, referred further as NED). The Down (D) component of the acceleration, *acc*_*D*_, is obtained from the VN100 at a frequency of 10 Hz and used for evaluating waves statistics. This 10 Hz value is obtained by applying a Kalman filter to the raw sensor output (that is obtained at several kHZ), which translates into a 800 Hz processed output. After that, a running average over 80 samples is performed to obtain the final 10 Hz value. In the case when instruments are collected at the end of deployment, the full time series of the VN100 output at 10 Hz, is available on the internal SD card. This includes both raw sensor measurements, Kalman filter state, heading and orientation output, and acceleration in the Earth frame of reference.
- In the case of the v2021^38^, the temperature-compensated 9-degree-of-freedom (9dof) sensor measures the accelerations, angular rates, and the local magnetic field, each over 3 axis (the X, Y, and Z axis of the 9dof sensor, in its own frame of reference attached to the microchip itself). These raw measurements are performed at 800 Hz. Averaging and n-sigma filtering over 8 raw samples is used to downsample the signal from 800 Hz to 100 Hz, reject possible outliers, and reduce noise. An open source Kalman filter implementation is then used to fuse the data at 100 Hz update frequency. The Kalman filter produces a unit quaternion estimate *q* of the absolute orientation of the sensor relative to the Earth frame of reference. This information, combined with the acceleration measurements *acc*_*refSensor*_ = [*acc*_*X*_*, acc*_*Y*_*, acc*_*Z*_] in the sensor frame of reference, allows to retrieve the acceleration of the sensor in the Earth frame of reference *acc*_*refEarth*_ = [*acc*_*N*_*, acc*_*E*_*, acc*_*D*_], by applying the rotation described by the quaternion *q* on *acc*_*refSensor*_. At present, magnetometer calibration is not performed carefully enough to trust directional information relative to the magnetic North direction (this is ongoing work for further deployments). Therefore, the only data used at the moment are the vertical accelerations. These are averaged from the 100 Hz *acc*_*D*_ output into a 10 Hz wave acceleration signal.
- Similar to the v2018 instrument, the IWR uses the Vectornav VN100 IMU. Processing and settings similar to the ones used with the instrument “v2018” are used by the IWR. However, the IWR records wave acceleration continuously, and stores it on-the-fly on an attached SD card, so that the time series of the wave displacement are available. Initial deployments were storing every 80^*th*^ value at the rate of 10 Hz. Later, to improve data quality, the sensors were reprogrammed to average sequences of 80 values, which were then saved at 10 Hz. Data output differed between the deployments, but always included [*acc*_*N*_, *acc*_*E*_, *acc*_*D*_] along with yaw pitch roll angles of the IWR. The accelerations were output with constant vertical gravity acceleration removed.

At this stage, the vertical acceleration, which is the superposition of the gravity and wave acceleration (except for the IWR, where gravity is removed), is available at 10 Hz for further processing in the case of either the v2018 or the v2021 (the IWR is a pure logger that is not equipped with iridium transmissions and does not perform *in-situ* processing of the data).

### Estimation of the wave spectrum

The 10 Hz wave acceleration component alongside the Down (D) direction in the Earth frame of reference is then used to compute the wave spectrum and its statistics. For this, two variants of the same core methodology are applied in the case of the instrument v2018 and v2021.

- In the case of the instrument v2018, details of the methodology are presented in the technical paper describing the instrument^37^. We reproduce the main lines of the processing here. First, the vertical acceleration is integrated twice in time using the methodology of previously developed instruments^49^, which is done in Fourier space by using the frequency response weights of 1/*ω*^2^ and a half-cosine taper for the lower frequencies to avoid an abrupt cut-off^50^, corresponding to:
  1 $$\eta (t)=Re\left(IFFT\left[H(f)FFT(ac{c}_{D})\right]\right),$$
  where *Re* indicates the real part of the signal, IFFT stands for the Inverse Fast Fourier Transform, FFT the Fast Fourier Transform, and *H*(*f*) is the half-cosine taper function:
  2 $$H(f)=\{\begin{array}{cc}0, & 0 < f < {f}_{1}\\ \frac{1}{2}\left[1,-,{\rm{c}},{\rm{o}},{\rm{s}},(\pi \frac{f-{f}_{1}}{{f}_{2}-{f}_{1}})\right]\left(\frac{-1}{2\pi {f}^{2}}\right), & {f}_{1}\le f\le {f}_{2}\\ \frac{-1}{2\pi {f}^{2}}, & {f}_{2} < f < {f}_{c},\end{array}$$
  where *f* is the frequency, *f*_*c*_ is the Nyquist frequency, and *f*_1_ = 0.02 Hz and *f*_2_ = 0.03 Hz are the half-cosine taper corner frequencies, similar to^49^.
  Following the calculation of the time series for the wave elevation *η*(*t*) at 10 Hz, the wave elevation spectrum *S*(*f*) is estimated using the Welch method on 12000 samples (20 minutes at 10 Hz), using a Hanning window of length 1024 samples per segment, and 50% overlap. In addition, the spectral moments *m*_0_, *m*_1_, *m*_2_, *m*_4_ of the wave spectrum are computed, following:
  3 $${m}_{n}={\int }_{0.05}^{0.25}{f}^{n}S(f)df.$$
  This allows to compute the usual wave statistics, i.e. the significant wave height calculated from the time series, *H*_*st*_ = 4*std*(*η*), the significant wave height calculated from the spectral moment ${H}_{S0}=4\sqrt{{m}_{0}}$, the spectra peak period *T*_*p*_ corresponding to the maximum of the spectrum, the zero-upcrossing period ${T}_{z}=\sqrt{{m}_{0}/{m}_{2}}$, and the average crest period ${T}_{c}=\sqrt{{m}_{2}/{m}_{4}}$.
  In addition to these data, some directional spread estimates are computed, though these are not as carefully validated and their accuracy may be lower. The reader who wants to use these is invited to read about the technical details in the technical paper^37^, and to implement their own quality checks if they want to use directional information.
- In the case of the instrument v2021^38^, a slightly simpler methodology is used. The Power Spectral Density (PSD) for the vertical wave acceleration *PSD*_*accD*_ is directly calculated from the vertical wave acceleration data at 10 Hz, by applying the Welch method on 20.48 minutes of data (so that the exact number of samples is a multiple of 2^11^, which makes the calculation of the FFTs and the splitting into segments simpler and faster). Each segment for the Welch method has a length of 2048 samples, 75% overlap is used between the segments, and an energy-preserving Hanning window is used.

From there, the spectrum for the wave vertical elevation *S*(*f*) can be retrieved as:

4 $$S(f)=\frac{PS{D}_{accD}(f)}{{\left(2\pi f\right)}^{4}}.$$

At this stage, the spectral moments, as well as *Hs*, *T*_*z*_, and *T*_*c*_, can be calculated in the same way as for the instrument v2018.

### Data transmission and decoding

Data are transmitted from the sea ice by the instruments v2018, v2021, and the Sofar Spotter, using the Iridium communication network and the Short Burst Data (SBD) protocol, which allows messages of size up to 340 bytes.

- In the case of the instrument v2018, the wave spectrum *S*(*f*) is downsampled into 25 logarithmically equally spaced bins between 0.05 Hz and 0.25 Hz, and transmitted together with the wave statistical parameters.
- In the case of the instrument v2021, the full PSD of the wave vertical acceleration *PSD*_*accD*_(*f*) between 0.05 Hz and 0.3 Hz is transmitted, alongside with the wave statistical parameters, though these are merely a cross validation of the spectrum, in the sense that they can be derived directly from the transmitted full spectrum. A simple script, which performs no further processing of the data, is then used to decode the binary SBD messages and apply the translation from *PSD*_*accD*_(*f*) to *S*(*f*) following Eqn. 4.
- In the case of the Sofar Spotter, a variety of modes are available, in which either the full spectrum, or only integrated quantities, are transmitted. The data are available as JSON files, which are provided directly by Sofar by unpacking the binary data.

Commercial iridium trackers provide the track information data through a web-based API that abstract the details of the data transmission and processing protocol.

## Data Records

All our high level, processed data are provided as netCDF4-CF files on the THREDDS data server of the Norwegian Meteorological Institute through the Arctic Data Center (ADC) database, at the following address (which references the individual deployments and files, each of them containing more detailed metadata): https://adc.met.no/datasets/10.21343/azky-0X44^51^. Extensive metadata are provided, so that the different data fields are self documenting, following the FAIR data principle^52^. In addition, all the raw iridium data transmissions (hex-string binary data), and scripts for reading these raw data files and processing them into netCDF files, as well as example scripts of how the netCDF datasets can be read, are available on Github in the folder structure of the following repository: https://github.com/jerabaul29/data_release_sea_ice_drift_waves_in_ice_marginal_ice_zone_2022.

In the following, we provide an overview of the different deployments, what kinds of instruments and how many of them were used in each of these, and some background information about the sea ice conditions and the data of interest. As there are too many buoys and deployments involved, it is not feasible to provide a per-buoy description of the data here, nor to illustrate every individual deployment. The reader who wants a quick overview of the data available through figures displaying what information is available from each deployment, is referred to the github repository https://github.com/jerabaul29/data_release_sea_ice_drift_waves_in_ice_marginal_ice_zone_2022/tree/master/Data, and the figures therein. The time series obtained are of variable duration and some of them have holes in the data. This is due, to the best of our knowledge, to the harsh conditions found on sea ice, rather than technical issues with the instruments. Factors such as heavy snowfalls, polar bear destroying equipment, sea ice breakup, ridging and rafting, are all susceptible to interrupt iridium communications or destroy prematurely the instruments altogether.

The deployments in the Arctic are, in chronological order:

- 2017-04: Arctic deployment in the MIZ that contains drift data in the Barents Sea. 8 GPS ice trackers were deployed on 6 ice floes in the Barents Sea South of Svalbard. The trackers send GPS data every 30 minutes, and were deployed during April 24–26 2017 from the Research Vessel Polarsyssel. Trackers 4610 and 8650 were deployed on the same ice floe, and trackers 5630 and 2470 were deployed on the same ice floe, different from the previous one. The other trackers were deployed each on their respective ice floe. An overview of the deployment time for the different trackers is provided in Table 1. This dataset has been described and analyzed in details in^53^. We only summarize the main lines of the corresponding study here, and we refer the reader curious of more details to the full study^53^. The trackers could float and, therefore, the internal temperature records were used to determine the date and time when they entered water, as the temperature transitioned from a larger diurnal cycle when exposed to air on top of the ice, to a significantly more muted diurnal temperature variation when floating in open water. An analysis of the trackers’ internal temperature records and the Svalbard regional daily ice maps from the Norwegian Meteorological Institute Ice Service led to the determination that the beacons most likely began falling into open water sometime on May 2, 2017^53^. The dispersion of the ice floes was dominated by strong shearing within the local ice pack, coinciding with a rapid increase in the speeds of the local tidal currents, which was soon followed by a rapid increase in the wave energy. Each of the six tracked ice floes increased their observed drift speeds in sync with the increase in the local tidal current speeds at different times for each floe, but at approximately the same decrease in water depth as they reached the northern edge of Spitsbergen Bank. The rapid increase in the tidal currents was linked to the topographic enhancement of tidal motion near Hopen Island in the shallower waters of Spitsbergen Bank. The last transmission from the trackers was received 2017-07-15.

**Table 1:** Details around the deployment conditions for the 8 ice trackers, Svalbard Banks, 2017-04.

Tracker ID	deployment time (UTC)	floe dimensions (m)	floe surface area (m^2^)
4610	April 24 2017, 08:54	30 × 43	934
9630	April 25 2017, 07:59	10 × 25	250
2620	April 25 2017, 08:05	10 × 15	150
3620	April 25 2017, 08:10	5 × 8	40
0630	April 25 2017, 08:15	20 × 30	600
2470	April 26 2017, 08:20	10 × 25	250
- 2018-03a: Arctic deployment in the MIZ, in the East Greenland Sea. The primary aim of the cruise was to monitor the production of seal pups. For that, 5 GPS and iridium trackers were deployed on large ice floes in the dense MIZ and drifted Southwestwards, following the East Greenland Current. GPS data sampling rate was 30 minutes. The trackers were deployed between March 20th, 2018, and March 23rd, 2018, and the last tracker stopped transmitting on April 25th, 2018, though communications were unreliable (meaning that some messages were lost, but the content of messages are checksum-validated by the iridium protocol itself, therefore, if a message goes through, its integrity can be trusted) after April 6th, 2018, possibly indicating that the trackers were covered by snow and ice. The trackers were not equipped with floatability equipment, so that the trackers are guaranteed to be on an ice floe for all the trajectory duration. More information is available in^54,55^.
- 2018-03b: Arctic deployment on pack ice in the Beaufort Sea during ICEX2018, U.S. Navy exercises. Two IWRs were deployed for 4 days near an ice camp on 2018-03-17. One instrument (IWR2) was located on level ice near the air strip. It recorded numerous events characterized by strong, high-frequency oscillations produced by planes landing and taking off. The events matched the camp’s logbook. The second instrument was placed in an ice rubble field nearby. It also measured several cases of strong accelerations, not coinciding with the first instrument observations. Their origin is unclear. The instruments were recovered on 2018-03-21. More information is available in^56^.
- 2018-04: Arctic deployment in the MIZ and drift ice in the Barents Sea. An ice tracker by Oceanetic Measurements Ltd was deployed on an ice floe to monitor the floe drift. GPS location was sent with an interval of 10 minutes. The tracker was deployed on April 27th, 2018 and transmitted until February 27th, 2019. The tracker drifted in the region of the Spitsbergen Bank for approximately 6 months, though it was on an ice floe only until around May 3rd, 2018 according to ice charts from cryo.met.no, and after this date, the tracker was floating in ice-free waters. More information are available in^57^.
- 2018-09: Arctic deployment in the MIZ in the Barents Sea, in the context of the Nansen Legacy project, Physical Process Cruise 2018. In this deployment, a total of 4 instruments v2018 were deployed while the icebreaker R/V Kronprins Haakon was traveling into the ice. The first instrument was deployed on a lone ice floe in the outer MIZ, while sea ice concentration (SIC) was about 1/10. The second instrument was deployed on an ice floe in dense drift ice, SIC about 5/10. The third instrument was deployed at the very start of the close pack ice, SIC about 9/10. The fourth instrument was deployed in the close pack ice, SIC about 10/10. All instruments were deployed on 2018-09-19. The cruise report is available if more details are needed^58^. The instruments worked for around 2 weeks. However, a strong storm took place around 2018-09-24, and after this time, the spectra reported by the v2018 are very noisy with a lot of high frequency wave energy. This indicates that the supporting ice floes broke into small pieces and started to drift in very open water, which is confirmed by SIC from models (see, for example, the discussions in manuscripts using the data^26^). Therefore, the GPS drift tracks can be trusted over the whole duration of the dataset, but spectra dominated by high frequency signal correspond to small, broken ice floes drifting in very open water and experiencing erratic oscillations, rather than more classical waves in ice.
- 2020-03a: Arctic deployment on landfast ice in Grønfjorden, Svalbard. Three v2018 were deployed along the main axis of the fjord on continuous landfast ice between 10 and 13 March 2020. The instruments were deployed at a distance of 500 m, 1100 m and 1800m from the unbroken ice edge (i.e., the start of the continuous landfast ice), respectively. At the time of deployment, the unbroken sea ice covered approximately half the length of the fjord (which is 16 km long in total). The other half was covered by broken ice and extended up to the entrance of the fjord. Ice thickness was measured at various locations near the instruments between 30 and 40 cm. Instruments were recovered after approximately two weeks on the 28th of March, 2020. At the time of retrieval all instruments were still deployed on unbroken ice and recording data. These data were used in some previous works^42,59^, and some additional information are available there.
- 2020-03b: Arctic deployment on pack ice in the Beaufort Sea during ICEX2020. The deployment strategy was similar to the ICEX2018 campaign (see 2018-04). Deployment took place on 2020-03-08, and the instruments were collected on 2020-03-18. Unlike the previous ICEX deployment, the instrument close to the air strip (IWR6) did not capture events associated with landing planes. No logging of arriving and leaving planes was made. Both IWRs recorded two events of propagating flexural-gravity waves presumably forced by strong winds during passing storms. Figure 3 shows a marked difference between signals created by planes and associated with propagating flexural-gravity waves. More information can be found in^56^.
- 2020-07: Arctic deployment in the MIZ in the Barents Sea over the Yermak Plateau, in the context of the Fram-2020 hovercraft expedition. The cruise report is available at^60^ In this deployment, a total of 6 instruments v2018 were deployed over the summer 2020 on drift ice, SIC ranging from approximately 3/10 to 10/10. One instrument was deployed 2020-07-15, and transmitted until 2020-07-31. One instrument was deployed 2020-07-21, and transmitted until 2020-09-03. However, the energy content in the high frequency part of the spectrum of this instrument indicates that it likely drifted on a small, isolated, broken ice floe from around 2020-08-19, as illustrated in Fig. 2. One instrument was deployed 2020-08-14, and transmitted until 2020-09-08. Finally, 3 instruments were deployed around 2020-08-26, and transmitted until around 2020-09-24. Several wave events were consistently observed across the array of instruments deployed simultaneously, and clear wave damping is visible.

**Fig. 2:**
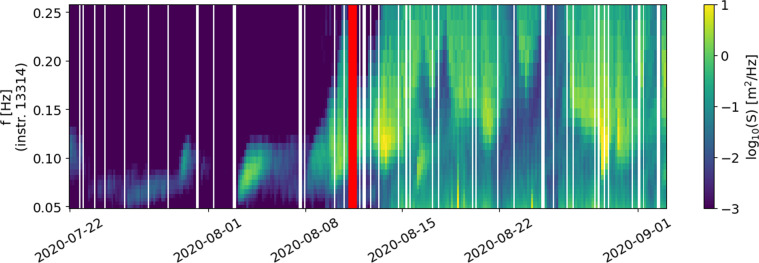
Illustration of the change in the wave elevation spectrum typical of the transition from pack ice or close ice conditions (left of the red line) to either small scattered ice floes or freely floating conditions (right of the red line). When an ice buoy is on pack ice or close ice, the ice acts as an effective high frequency filter, and the spectra show a sharp decrease of the energy content as the frequency increases. By contrast, a freely floating buoy or a buoy on top of a small, isolated ice flow, experiences a lot more high frequency motion, corresponding to short waves. This is easily distinguishable by the eye, as visible in the present figure.
- 2021-02: Arctic deployment in the MIZ in the Barents Sea, East of Svalbard, in the context of the Nansen Legacy project, PC-2 Winter Process Cruise 2021. In this deployment, a total of 6 instruments v2018, and 11 prototypes of the instrument v2021 (of which 6 were equipped with wave measurements) were deployed from the icebreaker R/V Kronprins Haakon on its way up and down the East coast of Svalbard, in close drift ice. The details of the deployment are reported in the cruise report^61^. 3 instruments v2018 were deployed on the way into the ice, for SIC increasing from around 5/10, 9/10 and 10/10. All the other instruments were deployed in close pack ice, for SIC of 10/10. The instruments gradually stopped transmitting as ice broke up, with the last transmission on ice taking place in late April.
- 2021-03: Arctic deployment on broken pack ice in the Beaufort Sea to accompany measurements during Sea Ice Dynamic Experiment (SIDEx2021). The instruments were deployed on 2021-03-06. In total six IWR instruments were installed surrounding the ice camp, but only three were recovered. Due to battery problems, the amount of collected data varied. The shortest observational interval was 2 weeks and the longest one and a half month (it was collected on 2021-04-22). More information is available in^56^.
- 2021-04: Arctic deployment on landfast ice near Utqiagvik, Alaska as a part of Integrated System for Operations in Polar Seas project. Six buoys were placed in different locations on landfast sea ice. The deployment took place in late April-early May of 2021. The operation period of the IWRs varied. Some instruments were collected earlier to prevent instrument and data loss in cases of worsening ice stability. Ice conditions varied between rubber fields and level ice of a refrozen lead. Ice thickness ranged between 0.6 m to several meters with areas of smooth ice being about 1 m thick. All buoys were located in the deformed ice. IWR 35 was distinguished by being placed behind an iceberg frozen into the lead. IWRs 30&33 were the closest to the landfast ice edge. The measurements did not exhibit any clear sign of waves. However, a wave event was detected via ground-based radar interferometry positioned on the iceberg. Respective vertical accelerations above IMU’s noise level were also found in the IWRs (e.g., IWR33). More on the deployment and results can be found in^62^.
- 2021-09: Arctic deployment in the MIZ in the Laptev Sea, in the context of the NABOS campaign. Two buoys, one Sofar Spotter and one instrument v2021 packed in a Zeni floating enclosure, were deployed adjacent to the ice edge on 2021-09-15. The Sofar Spotter battery life at these latitudes and without solar input is only around 10 days, but by contrast, the v2021 instrument functioned for over 2 weeks. Several swell events were measured during the deployment, and the distance between the buoys allows to observe clear attenuation of the incoming swells by sea ice. The data are used in^63^, and the reader is referred to the corresponding work for further details about the deployment and the data collected. The last transmission included in the dataset took place on 2021-09-29.
- 2022-03: Arctic deployment in the MIZ in the East Greenland Sea, in the context of the seal pup monitoring cruise 2022. In this deployment, 2 instruments v2021 were installed on 2 separate, neighboring medium size drifting ice floes (typical floe size: 25 m; typical floe thickness: 1.5 m) from the icebreaker R/V Kronprins Haakon on March 27th, 2022. In addition, 5 commercial GPS and iridium drifters that only measured sea ice drift were deployed on large ice floes in the dense MIZ (SIC around 9/10). The instruments drifted following the East Greenland current. The trajectories of the 2 instruments v2021 remained close (less than typically 2 km apart) for around 2 weeks, before drifting slightly further apart from each other but heading in the same general direction. As a consequence, tracks and wave spectra are initially very similar, before clear differences in the wave spectra due to being at different depths in the MIZ get visible after around 2 weeks of drift. One of the instrument started to transmit data unreliably after around 4 weeks of activity, likely due to being covered by a layer of snow and ice that blocked iridium communications (since occasional transmissions were still recorded after messages started to come in unreliably, which did not indicate any technical issue with the instrument other than bad communications). The second instrument worked uninterrupted for over 4.5 weeks. The trajectories of the 5 commercial gps and iridium drifters remained close for most of the deployment. The last transmission was received on 2022-05-22. More information is available in the cruise report^64^.

The deployments in the Antarctic are, in chronological order:

- 2020-01: Antarctic deployment on landfast ice on the eastern rim of the Amery Ice Shelf. Four instruments, two v2018 and two Sofar Spotter, were deployed along a transect perpendicular to the unbroken ice edge on 7 December 2019. The first Spotter was deployed 100–200 m from the unbroken ice edge, while a v2018 and the second Sofar Spotter (40 m apart from each other) were deployed at a distance of about 3.7 km from the edge, and one more v2018 was deployed about 9.3 km from the unbroken ice edge. At the time of deployment, the ice was measured between 1 and 1.2 m thick. No wave events or drift was recorded until the 2nd of January 2020 when a large section of the fast ice broke and started drifting northward. The first v2018 stopped transmitting on 22 January 2020, while the two Sofar Spotters stopped transmitting on 1 February 2020 (although one reconnected for half a day on 3 March 2020). The last message successfully transmitted by the final v2018 was on 10 March 2020. These data were used in papers^42,45^, and some additional information are available there.
- 2020-11: Antarctic deployment on landfast ice north of Casey Station. Deployment consisted of two v2018, deployed 1.9 km apart in October 2020. Instruments were recovered after about 3–4 weeks. Ice thickness was measured 1.1 m thick during the deployment and 1.3 m during retrieval. As the deployment site was separated from the Southern Ocean open water by roughly 300 km of broken sea ice, only limited wave energy was observed. The sea ice at the deployment site remained unbroken throughout the deployment. More information is available in^24^.

A summary of the deployments is provided in Table 2, and in Fig. 4.

**Table 2:** Overview of the deployments, their locations and time spans, the sea ice conditions, and the kind and number of instruments deployed.

Deployment time	location	ice conditions	number & kind of instrument
2017-04	Arctic, Barents Sea, 76.4 N 22.5E	drift ice: 8/10 to 0/10	GPS drifter: 8
2018-03a	Arctic, East Greenland Sea, 73.5 N 15.5E	drift ice: 6/10 to 10/10	GPS drifter: 5
2018-03b	Arctic, Beaufort Sea, 72.3 N 148.4 W	pack ice: 8/10 to 10/10	IWR: 2
2018-04	Arctic, Barents Sea, 75.3 N 19.5E	drift ice: 8/10 to 0/10	GPS drifter: 1
2018-09	Arctic, Barents Sea, 82 N 20E	MIZ: 1/10 to 10/10	v2018: 4
2020-01	Antarctic, outside Davis station, 69 S 76E	landfast ice (breakup)	v2018: 2 + Sofar Spotter: 2
2020-03a	Arctic, Grønfjorden, Svalbard, 78 N 14E	landfast ice (intact)	v2018: 3
2020-03b	Arctic, Beaufort Sea, 71.2 N 141.5 W	pack ice: 8/10 to 10/10	IWR: 2
2020-07	Arctic, Yermak Plateau, Barents Sea, 82 N 15E	MIZ: 3/10 to 10/10	v2018: 6
2020-11	Antarctic, outside Casey station, 66 S 110E	landfast ice (intact)	v2018: 2
2021-02	Arctic, Barents Sea, east Svalbard, 77 N 30E	MIZ: 5/10 to 10/10	v2018: 6 + v2021: 11 (6 with waves)
2021-03	Arctic, Beaufort Sea, 71.5 N 148WE	pack ice: 7/10 to 10/10	IWR: 3
2021-04	Arctic, Utqiagvik, 71.3 N 156.6 W	landfast ice	IWR: 6
2021-09	Arctic, Laptev Sea, 82 N 118E	MIZ: 1/10 to 10/10	v2021: 1 + Sofar Spotter: 1
2022-03	Arctic, East Greenland sea, 70 N 20E	MIZ: 2/10 to 10/10	v2021: 2 + commercial beacon: 5
total nbr tracks			total: 72; with waves: 48

**Fig. 3:**
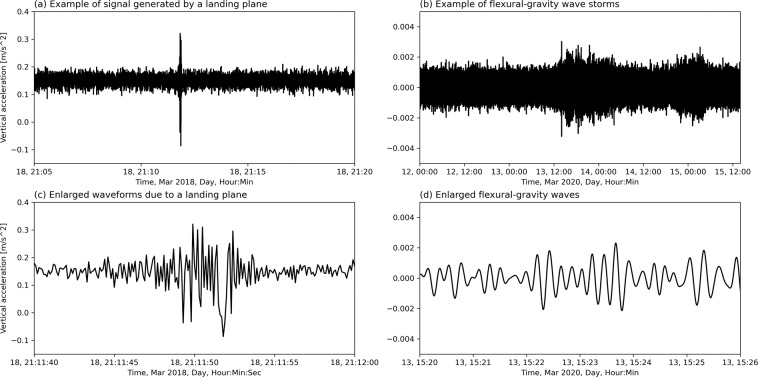
Illustration of the typical signal shapes obtained when planes are landing (**a**,**c**), vs. when waves are present (**b**,**d**). At a coarse time scale, displaying only the typical amplitude of the signals recorded, plane landing is typically visible as a short burst of very intense vertical motion (**a**), while wave signal shows a smaller increase in the signal amplitude, which takes place over a longer time (**b**). When looking into the small scale signal features, plane signal typically contains very high frequencies that likely arise from aliasing of propeller vibrations. This translates into a signal that quickly jumps between high and low values from reading to reading (see panel (**c**): over the 5 seconds from 21:11:45 to 21:11:50, there are only 50 measurement points, and the signal oscillates quickly from reading to reading). By contrast, when waves are present, a smooth wave signal with much lower dominating frequency (typically 0.1 to 0.05 Hz) is visible, as shown in panel (**d**).

**Fig. 4:**
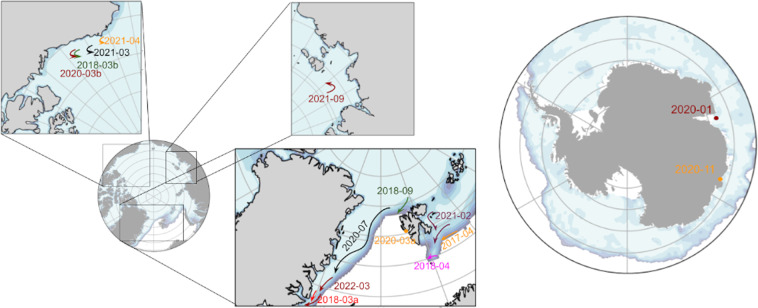
Overview of the deployments present in the dataset. The SIC map show the averaged SIC over the local winter month in the Arctic and Antarctic.

## Technical Validation

There is no need for validation regarding GPS position measurements, as these are well established sensors with a well known accuracy (typically +−5 m in good signal conditions according the the datasheet).

Regarding wave data, we are only using well established, longstanding methodologies. The sensors used are thermally calibrated over a range that typically exceeds the range of conditions found in the field in the MIZ (the Vectornav VN100 used in the v2018, is thermally calibrated over the full temperature range from −40 to + 85 C, while the ST-Microelectronics ISM330DHCX used in the v2021 is calibrated from −40 to + 105 C, according to their respective datasheets). Therefore, we consider that the data acquisition for the wave acceleration by itself does not need additional validation, and users should refer to the datasheets of the corresponding sensors for further information. As a side note, validation of the accelerometer data was performed, either directly (see Fig. 1 of^46^ for the test and validation performed for the VN100), or indirectly (by validating the accuracy of wave spectra, see next paragraph). In practice, however, raw data from the instrument are only available for a couple of deployment of the instruments v2018 (when the SD card could be recovered), and for the IWR deployments, for which the data provided are always the timeseries of the IMU output.

**Fig. 5:**
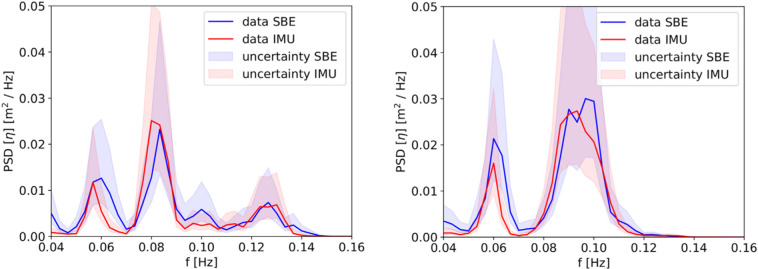
Validation of the instrument v2018 IMU measurement and processing (IMU curves) against co-located pressure-sensor-based measurements of waves using a Seabird pressure sensor (SBE curves), reproduced from ^37^. The measurements were obtained during a test campaign of the IMU measurement methodology performed on landfast ice in Tempelfjorden, Svalbard, in 2016. Shaded areas indicate the 5-sigma confidence intervals, computed from the Chi-squared uncertainty distribution estimate for stochastic Gaussian wave spectrum following the methodology discussed in^80,81^. Agreement within the confidence intervals is observed.

**Fig. 6:**
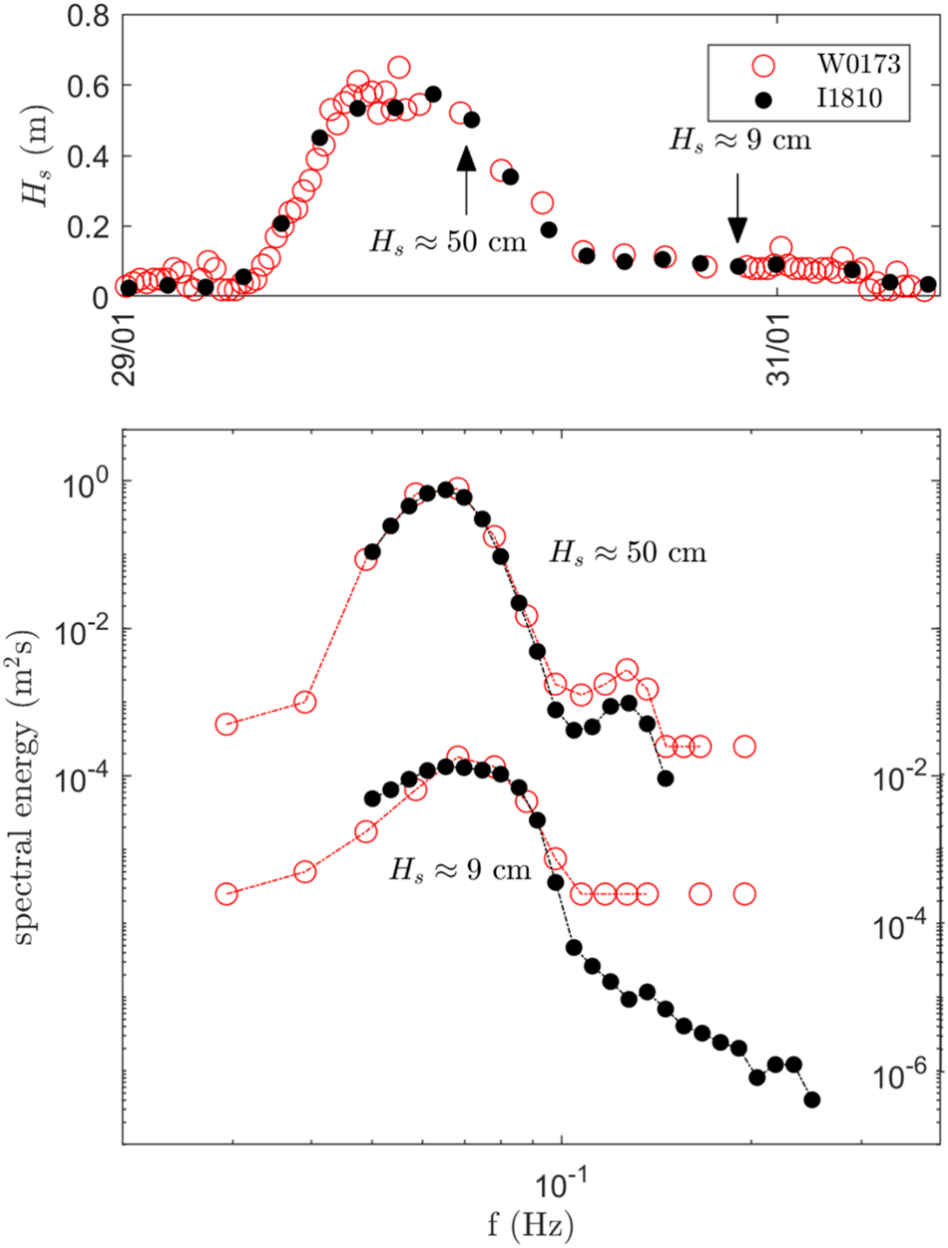
Validation of the instrument v2018 (I810) against the Sofar Spotter instrument (W0173), reproduced from^82^. The instruments were deployed close to each other, but drifted slightly with time. Still, agreement within 5% is obtained for the significant wave height estimate (Hs, top), and the location and height of the wave spectrum peak (bottom). Slight differences are observed regarding the high frequency tail of the spectra. This is due to the increased noise of the GPS-based measurement methodology of the Sofar Spotter in quiet conditions corresponding to waves in ice, compared with the accelerometer-based measurement methodology of the v2018 in the same conditions.

**Fig. 7:**
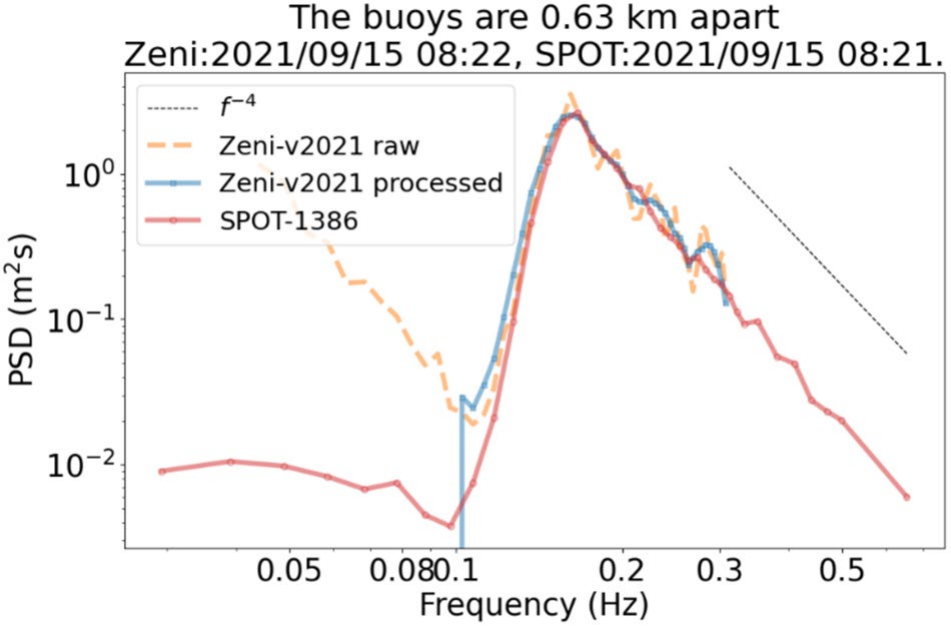
Validation of the instrument v2021 (Zeni-v2021 processed) against the Sofar Spotter (SPOT-1386), in open water, reproduced from^38^. The instruments were deployed close to each other, but drifted significantly with time. Agreement within typically 5% is obtained on most of the spectrum. The increasing, spurious noise at low frequencies observed in the Zeni-v2021, is a well known phenomena for IMU-based measurements of waves in ice in the open ocean (which results from the noise present in the Kalman filter attitude estimate under rough sea conditions, in combination with the intrinsic spectral noise background inherent to MEMS sensors, and the double integration corresponding to Eqn. (4); for more details, see^83^), which can be filtered out. This phenomenon is not expected to be present when performing measurements of waves in the sea ice.

**Fig. 8:**
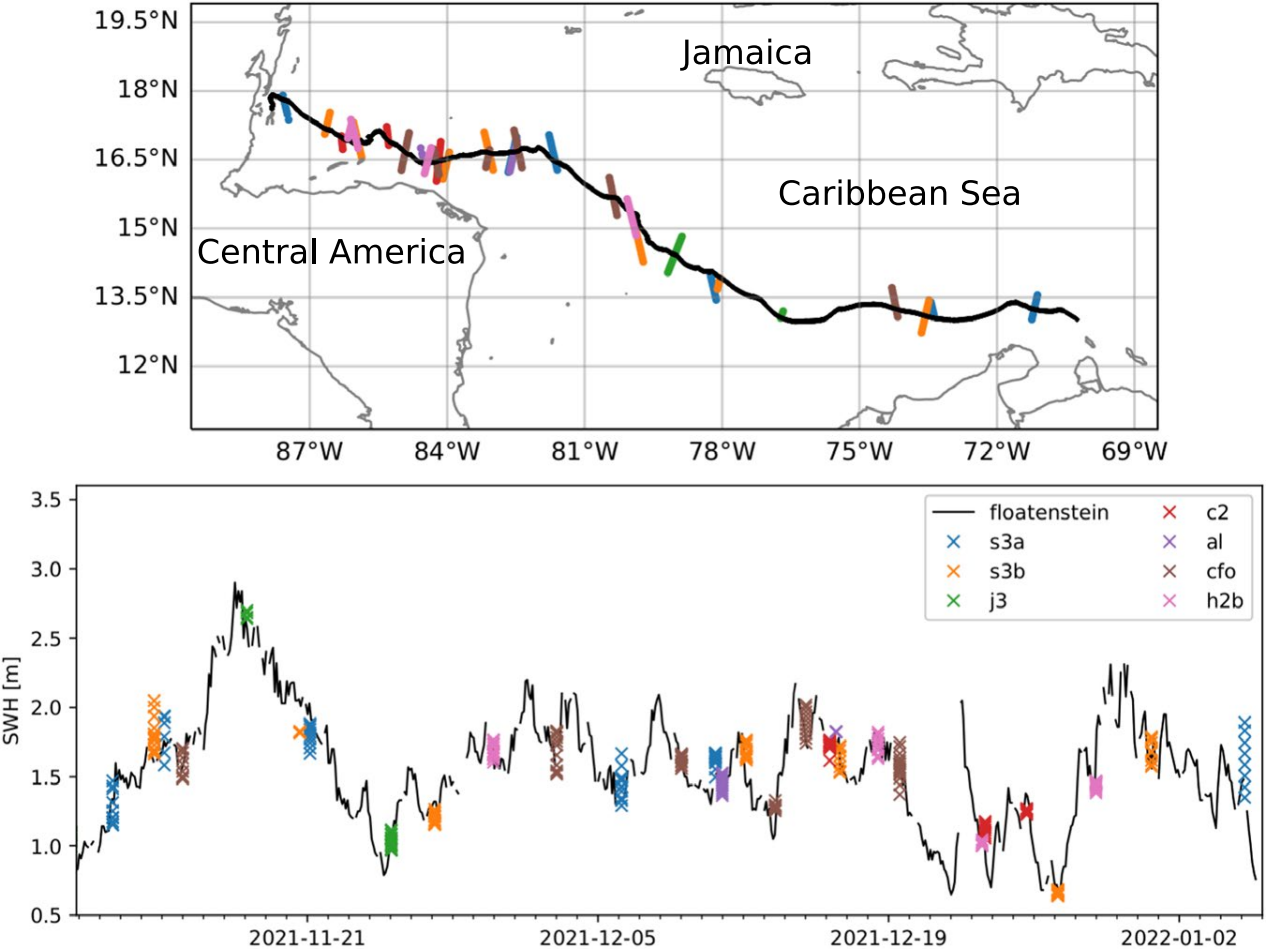
Validation of the instrument v2021 against satellite measurements, reproduced from^38^. Top: drift trajectory of a freely drifting, open water version of the instrument v2021 (black), and illustration of satellite swaths intersecting the trajectory over the drift period (colored point clouds). Bottom: comparison of the significant wave height (SWH) reported from the v2021 (nicknamed “floatenstein”), with satellite measurements from a variety of satellites. SWH comparison between the v2021 and the satellite measurements are in agreement, with the measurements from the v2021 always falling within the spread for each satellite measurement swath. While this is obtained from a deployment performed in the Caribbeans, this is a validation of the correct functioning of the wave measurement routines. The satellites used are: s3a: Sentinel-3A; s3b: Sentinel-3B; c2: CryoSat-2; j3: Jason-3: al: SARAL/ALtiKa; cfo: CFOSAT (on board altimeter not from the SWIM instrument); h2b: Hai Yang 2B.

**Fig. 9:**
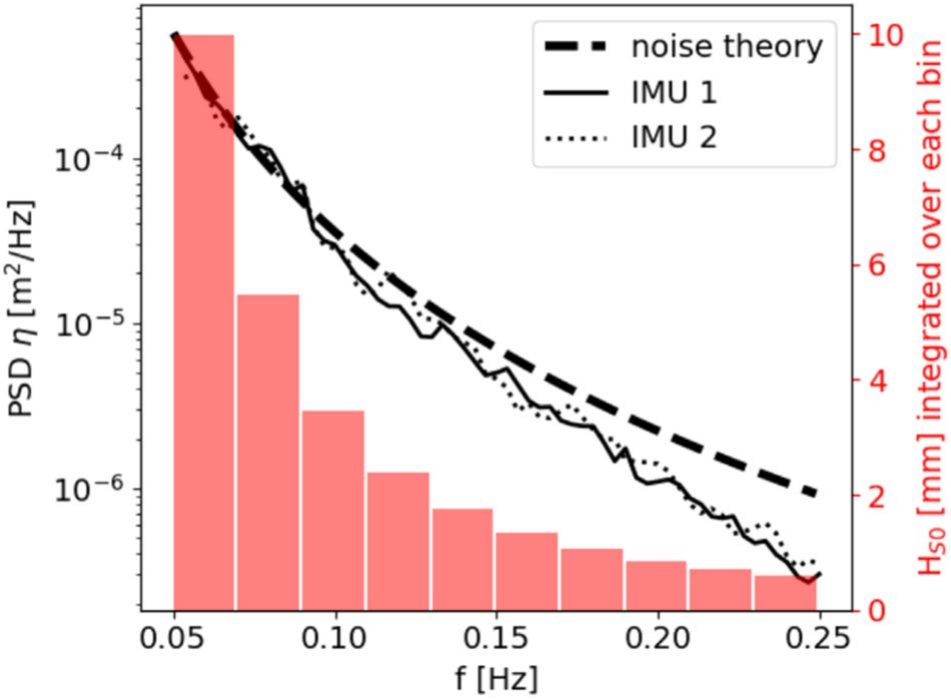
Illustration of the noise threshold at rest for the instrument v2018, reproduced from^37^. This characterizes the typical noise level of the combination of the VN100 IMU and the wave processing algorithm used to generate the spectra transmitted over Iridium.

**Fig. 10:**
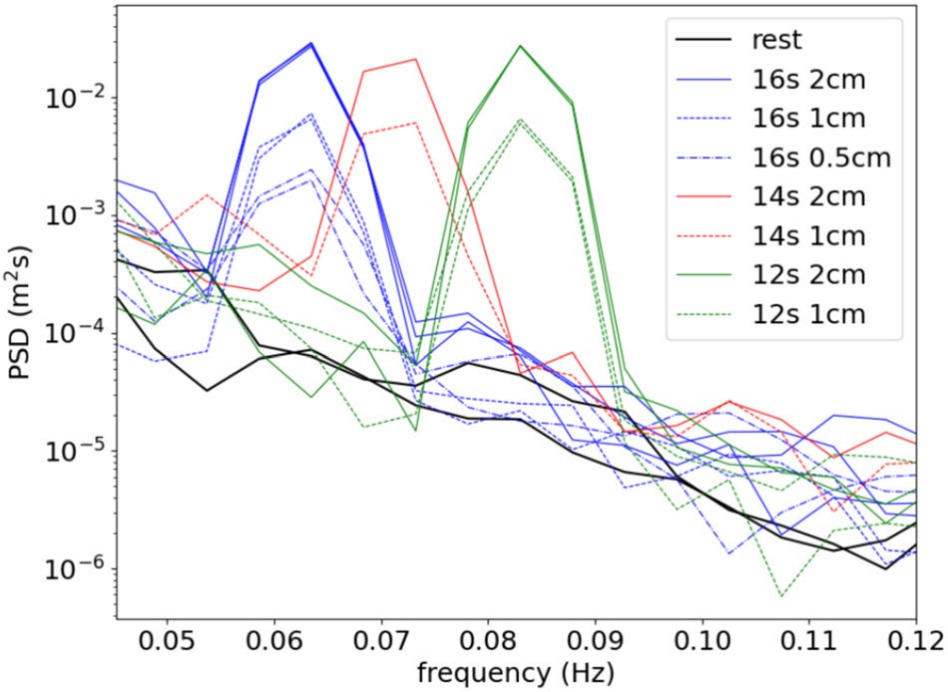
Illustration of the noise threshold at rest and under a variety of wave motions produced artificially in the laboratory for the instrument v2021, reproduced from^38^. This characterizes the typical noise level resulting from the combination of raw data measurements by the accelerometer and gyroscope, and the processing algorithms used.

Results that come from *in-situ* processing of IMU or GPS data, such as the wave spectra and statistics reported by the Sofar Spotter and the instruments v2018 and v2021, have been previously validated in detail, and the reports and validation details are available in the literature: the Sofar Spotter is a commercial instrument that was validated before being released for sale^40^, the v2018 has been validated and used in scientific papers multiple times^37,42,59^, and the v2021 has been recently validated against both commercial buoys and satellite data^38^. Validation campaigns for both the v2018 and the v2021 indicated agreement to either within 5%, or within one standard deviation, of commercial instrument or other measurement methodology. While more details are available in the corresponding papers, we reproduce the main validation figures against established commercial instruments in Figs. 5, 6 (v2018) and Figs. 7, 8 (v2021), for the sake of completeness. While Fig. 8 corresponds to validation in the open ocean (“floatenstein” floating buoy), these are actually more demanding conditions from a signal processing point of view, compared to what is obtained in the sea ice. This is true in particular for the Kalman filtering, as lots of random high frequency motions are present in the “floatenstein” case given the shape of the drifter, compared to what is obtained in the sea ice. Therefore, this validation, together with the actual validation in the MIZ from Fig. 7, helps us demonstrate the robustness of our implementation. In addition, since the v2018 and the v2021 have much higher levels of sensitivity compared with GPS-based buoys or satellite measurements against which we validated them, tests were performed in the laboratory, in controlled conditions, in order to estimate the noise background of the whole system (i.e., including the noise of the IMU itself, and the effect of the processing algorithms used, as described in the Methodology section). As visible in these tests, which main findings are reproduced in Fig. 9 (v2018), and Fig. 10 (v2021), the instruments are able to measure waves with amplitude down to typically a few millimeters. Test results are in agreement with the accelerometer datasheet (which describe the expected noise background intensity), and the formula for conversion from acceleration to elevation spectrum (Eqn. 4, which describes the expected spectral shape of the noise), where the noise backgrounds of the v2018 and the v2021 follow the expected decay curve as frequency increases (i.e., accelerometer-based instruments are more sensitive for wave amplitude as wave frequency increases). The typical noise threshold for a single data bin for the instrument v2018 is about 10 mm for 20 s waves, and 0.3 mm for 4 s waves, as visible in Fig. 3 of^37^, reproduced here as Fig. 9. The typical noise threshold for the v2021 is even lower, as visible in the Fig. 5 of^38^, reproduced here as Fig. 10, as a 16 s wave of amplitude 5 mm corresponds to a signal to noise ratio (SNR) of around 10.

These validations, both against other field instruments, against satellite measurements, and in controlled laboratory conditions, give us confidence in the accuracy and reliability of our instruments. Moreover, a good understanding of the noise characteristics of sea ice buoys is critical to avoid mis-interpretation of the signals recorded, and over-interpretation of the underlying physics. This has been increasingly discussed in context of the analysis of high frequency rollover observations, which have recently been proven to possibly find their origin in either the noise characteristics of some of the buoys used^65^, or observations timing and wave propagation delay effects^66^. Our open source solutions allow full insight into the internals of the processing performed, which can help alleviate these difficulties.

## Usage Notes

Two kinds of data processing levels are provided to the user:

- High-level data that are ready-for-use are provided as netCDF-CF files, following the best practices in use in the geoscience community and the FAIR principles^52^. These are available on a specific folder of the THREDDS server of the Norwegian Meteorological Institute as part of the Arctic Data Center repository 10.21343/AZKY-0X44^51^. Any netCDF package, in python or other language, can be used to access the data. The use of NetCDF4-CF files ensures long time compatibility, as this is the standard used for archiving meteorological data all over the world, and the tooling is mature and stable.
- In addition, the full raw Iridium transmissions are available at the Github repository that supports the data release https://github.com/jerabaul29/data_release_sea_ice_drift_waves_in_ice_marginal_ice_zone_2022. Custom software is provided (either on the same repository, or on the repositories where the source code of the instruments and post processing scripts are available) to decode these raw data. The fact that both the firmware of the instruments (hence, the binary protocol used), and the binary decoders, are open source, guarantees long term accessibility of the data, as well as full insight into the technical details associated.

No additional processing is needed to use either the GPS or the wave data, and the data are ready to use as-is in Python or similar.

More specifically, the netcdf files typically contain the following set of information (with minor variations between instrument kinds, and what data are collected):

- *lat* and *lon* fields, containing the latitude and longitude in degree decimal format,
- *frequency*, containing the list of frequencies corresponding to each bin of the spectra, in Hz,
- *trajectory*_*id*, corresponding to a unique identifier for each of the instruments present in individual netCDF files,
- *message*_*king*, which indicates if the entry with the corresponding index is a GPS fixes message (kind ‘G’), or a wave spectrum message (kind ‘W’). Only ‘G’ messages will contain a valid GPS fix (lat and lon), and only a ‘W’ message will contain a valid wave spectrum entry (*wave*_*spectrum* and other wave statistics),
- *wave*_*spectrum*, which contains the wave elevation spectrum at the bins of frequencies described in the frequency field. For the instrument v2018, these bins are downsampled from the actual Welch transform, meaning that to reduce the size of the messages, the transmitted frequencies are different from the actual Welch spectrum output frequencies. The downsampling from the Welch spectrum output to the downsampled output is performed by means of a trapezoidal integration. For more information about the details of the downsampling, see^37^. For the instrument v2021, the bins are a subset of the Welch transform output bins, and no downsampling is applied. The unit for the wave elevation spectrum is *m*^2^.*s* (also referred to as *m*^2^/*Hz*), which is the standard unit for such spectra.
- In addition, a number of wave statistics are computed *in-situ* by the instruments and transmitted over iridium in addition to the spectra. These quantities are redundant with the information available in the *wave*_*spectrum* variable, and are described in the metadata of the netCDF files. In particular, we transmit the significant wave height estimated from the wave elevation (instrument v2018 only) following the formula *swh* = 4*std*(*η*), where *η* is the wave elevation (computed on board by the instrument v2018), and estimated from the zeroth-order moment (see definition in Eqn. (3) $hs=4\sqrt{\left({m}_{0}\right)}$), as well as estimates of the wave period (see netCDF metadata). Note that all these estimates can be re-computed from the transmitted 1D spectra if necessary.

Examples of how these files can be read and plotted in the Python programming language are provided on the Github repository hosting the data, https://github.com/jerabaul29/data_release_sea_ice_drift_waves_in_ice_marginal_ice_zone_2022.

A proper use of the presented data for the analysis of wave damping will also critically depend on the availability of high-quality sea-ice concentration, thickness, and floe size distribution maps, as well as the wave frequency spectra in the open ocean adjacent to the sea-ice edge. While providing these data is, in general, outside of our scope, as there are a number of concurrent, different possible sources and models that produce such data, and these data are generated independently of the field measurements and rely on entirely different techniques, codes, and operational products, we highlight a few different sources that could be of interest to the reader.

Sea-ice concentration products are available from passive microwave sensors with a footprint of around 20 km^67^. New multi-sensor products are becoming available, combining sensor information of passive and active microwave and Synthetic Aperture Radar (SAR) sensors^68–70^. These products reach resolutions of the order of 1 to 10 km. In addition, manual sea-ice charts can be used which cover scales down to a typical order of magnitude of 100 m and are available on a daily basis from the national ice services (for example, the Norwegian Meteorological Institute Ice Service charts, https://cryo.met.no/en/latest-ice-charts, or the similar Danish Meteorological Institute Icecharts, http://ocean.dmi.dk/arctic/icecharts.uk.php). Various pan Arctic sea-ice thickness satellite products are available with resolutions of typically around 25 km and are considered reliable in the thickness range from 0.5 to 4 m^71^. Approaches to produce satellite based information on the sea-ice floe size distribution are emerging but to our knowledge no operational products are available yet^72^. Some national ice services (e.g. the Danish Sea Ice Service) include information on floe sizes as part of the manual ice charts^73^. In addition to the sea-ice information, wave information on the wave properties adjacent to the sea-ice edge are needed. A number of wave hindcasts and reanalysis are available, however, often the energy frequency spectra are not disseminated (e.g.^74^); we are currently providing this feedback to the modeling community, and hope that more detailed information from numerical models will be available in the future.

Regarding more specifically the possible uses of the data we release, these can be part of a number of studies about sea ice drift, waves damping by sea ice, and sea ice breakup. This has already been done, to some extent, by some of the authors and initial owners of some of the corresponding datasets. For example^42^, has adopted a “data first” approach to characterize specific waves and ice conditions that can lead to sea ice breakup^59^, has presented an in-depth analysis and validation of existing waves in ice damping models compared to field observations^26,57^, have presented case studies of sea ice drift properties and tuned models to *in-situ* observations^56^, has discussed observations of waves in ice deep into the continuous ice pack^63^, has analysed the shortcomings of existing sea ice models in thin ice conditions. In addition, we envision that these data can be used to further test, calibrate, and validate both sea ice drift and waves in ice models, as well as satellite processing algorithms, by serving as reference ground truth to compare against these methods.

## Data Availability

The firmwares of the instruments v2018 and v2021, as well as the binary data decoder scripts, are fully available on the corresponding github repositories: https://github.com/jerabaul29/LoggerWavesInIce_InSituWithIridium, https://github.com/jerabaul29/OpenMetBuoy-v2021a. The scripts to plot the data from the netCDF files are available on the main Github repository: https://github.com/jerabaul29/data_release_sea_ice_drift_waves_in_ice_marginal_ice_zone_2022, together with the raw data. All code is developed in modern python (version 3.8 or higher), or C++, or Matlab, unless specified otherwise. The netCDF datafiles are following the netCDF4 standard, with CF attributes conventions. We also provide a mirror of the netCDF data files on the THREDDS server of the Norwegian Meteorological Institute in the context of the Arctic Data Center repository, at the following address: 10.21343/AZKY-0X44. We will offer reasonable support regarding the data and its use through the Issues tracker of the data repository at https://github.com/jerabaul29/data_release_sea_ice_drift_waves_in_ice_marginal_ice_zone_2022, and we invite readers in need of specific help to contact us there. In addition, we plan on releasing extensions to this dataset periodically as more data are collected. We invite scientists who own similar data and are willing to release these as open source materials to contact us so that they can get involved in the next data release we will perform. In addition, we discovered, in the context of the present work, that there are already some openly available data about sea ice drift and waves in ice (for example,^75–77^, though there may be more such data available that we do not know of); however, these are scattered across the internet, and may be difficult to find. Therefore, in addition to the data release intrinsic to this dataset, we have started to maintain an index of similar open data at https://github.com/jerabaul29/meta_overview_sea_ice_available_data. We invite the reader aware of additional open datasets to notify us so that these can be added to our index, which we will keep extending in the future. We hope that these data, together with a variety of datasets that have been recently gathered^78,79^, will be a significant contribution towards building large, well sampled datasets of *in situ* observations of the MIZ and sea ice dynamics.
